# Mechanism-Based Neuromodulation in Augmenting Respiratory Motor Function in Individuals with Spinal Cord Injury

**DOI:** 10.3390/jcm14113827

**Published:** 2025-05-29

**Authors:** Farwah Fatima, Niraj Singh Tharu, Camilo Castillo, Alex Ng, Yury Gerasimenko, Alexander Ovechkin

**Affiliations:** 1 Kentucky Spinal Cord Injury Research Center, University of Louisville, Louisville, KY 40202, USA; 2 Department of Neurological Surgery, Division of Physical Medicine and Rehabilitation, University of Louisville, Louisville, KY 40202, USA; 3 Department of Medicine, Division of Pulmonary, Critical Care, and Sleep Disorders Medicine, University of Louisville, Louisville, KY 40202, USA; 4 Department of Physiology, University of Louisville, Louisville, KY 40202, USA; 5 Department of Neurological Surgery, University of Louisville, Louisville, KY 40202, USA

**Keywords:** neuromodulation, rehabilitation, respiratory motor function, spinal cord stimulation, respiratory muscles, respiratory muscle training, spinal excitability, neuronal plasticity, spinal cord injury

## Abstract

Spinal cord injury (SCI) is one of the most debilitating conditions that has profound effects on every physiological system, including respiratory dysfunction, which is listed among the most common causes of mortality and morbidity in this population. Previous research has demonstrated that respiratory training could facilitate respiratory motor- and autonomic activity-based plasticity. However, due to the reduced excitability of spinal networks below the level of injury, the effectiveness of such interventions is often limited to the residual functional capacity preserved after injury. In recent decades, several novel neuromodulatory techniques have been explored to enhance neuronal connectivity and integrate into respiratory rehabilitation strategies. In this review, we examine the mechanisms underlying respiratory deficits following SCI and discuss the neuromodulatory approaches designed to promote neural plasticity for respiratory recovery. Current evidence suggests that integrating multimodal neuromodulation with activity-based respiratory training holds promise; it may significantly enhance respiratory functional recovery and could become a standard component of respiratory rehabilitation protocols in individuals with SCI.

## 1. Introduction

Breathing is a fundamental physiological process that relies on the coordinated contraction of respiratory muscles, regulated by the respiratory motor control system [1]. Disruptions to this system, particularly due to injuries to the cervical and upper thoracic spinal cord, can significantly impair respiratory control, leading to respiratory dysfunction [2]. One of the most serious issues following spinal cord injury (SCI) is respiratory muscle weakness, which affects 36%–83% of patients during the acute phase and contributes substantially to respiratory failure in the chronic phase [1,3]. This dysfunction results in high morbidity and mortality rates in both the acute and chronic stages of SCI, with respiratory complications being a leading cause of death [4]. Notably, for every 1% decline in respiratory function, the risk of mortality increases by 3% [5].

Each year, approximately 17,000 new cases of SCI are reported in the United States [6], with around 2,300 cases involving upper cervical (C1–C4) injuries that require prolonged ventilatory support [7]. While mechanical ventilation is essential for sustaining life in these cases, it can also lead to adverse effects, such as altered venous return, an increased risk of respiratory infections, diaphragm atrophy, and reduced lifespan. Therefore, achieving ventilator independence is a primary therapeutic goal for individuals with SCI [7,8]. Despite advances in medical care that have improved survival rates, the mortality rate for individuals with SCI remains 47% higher than that of the general population [9]. Respiratory impairments in SCI vary widely, ranging from complete diaphragmatic paralysis in high cervical injuries to progressive restrictive changes affecting the respiratory muscles and bronchoalveolar structures in lower cervical and thoracic injuries [10].

Conventional respiratory management in individuals with SCI has primarily focused on achieving ventilation-independent breathing and reducing respiratory complications [11], aiming at strengthening the respiratory muscles. Additionally, neuromodulation techniques have emerged as promising interventions aimed at restoring and improving impaired respiratory functions following SCI [3]. Over the past several decades, neuromodulation has been extensively studied, demonstrating potential benefits in enhancing cough efficacy [12], respiratory muscle strength, and overall breathing function [13]. Although neuromodulation has been associated with respiratory neuroplasticity and improved breathing in individuals with SCI, the precise mechanisms underlying these effects remain unclear and warrant further investigation. Moreover, the implementation of neuromodulatory techniques in clinical practice poses challenges due to limited supporting evidence [14].

The objective of this review is to provide additional insights to support previous findings on respiratory neuroplasticity [2,15,16], while enhancing clinicians’ and researchers’ understanding of the mechanisms and potential of neuromodulation in augmenting respiratory rehabilitation. Despite receiving less attention compared with other rehabilitation strategies, respiratory neuromodulation has shown promise in both preclinical [17] and clinical studies [18], with evidence suggesting potential neuroplastic changes that can lead to functional motor improvements [19]. However, additional clinical trials are needed to assess the long-term effects of interventions targeting respiratory function. A comprehensive understanding of the pathophysiological mechanisms underlying respiratory dysfunction in SCI is crucial for identifying new therapeutic targets that could play a pivotal role in respiratory neurorehabilitation.

In this review, we explore the key components and neural circuitry of the respiratory network involved in normal breathing. We introduce the concept of the respiratory central pattern generator (R-CPG) as a general framework for understanding respiratory mechanics. While various studies have examined individual components of the R-CPG, the complexity of its interconnected pathways highlights the need for integrated research approaches. It is suggested that simultaneously stimulating multiple targets within the R-CPG may produce synergistic effects, enhancing neuroplasticity, improving respiratory function, and promoting long-term recovery—paralleling the findings from locomotor rehabilitation studies [20].

Furthermore, we review several neuro-rehabilitative interventions designed to stimulate the spinal cord, its pathways, and its associated musculature. Drawing on principles from locomotor studies, we present evidence of neuroplastic changes within the respiratory system and explore the interplay between respiratory function and locomotor coupling mechanisms. Although promising, further research is needed to confirm the efficacy of these interventions and facilitate their integration into standard clinical practice. This review focuses primarily on respiratory rehabilitative strategies, including spinal cord epidural stimulation (scES), respiratory muscle training (RMT), the respiratory–locomotor coupling phenomenon, and other approaches, including non-invasive spinal cord transcutaneous stimulation (scTS), providing evidence of the potential benefits of neuromodulation, offering valuable insights into its prospects in respiratory rehabilitation.

## 2. Concept and Mechanism of Respiration

### 2.1. Central Pattern Generator (CPG)

Central pattern generators (CPGs) are neural networks that generate rhythmic motor patterns, such as locomotion and breathing, independently of sensory or descending inputs [21]. Certain rhythmic motor patterns, like breathing, must be continually active, whilst others, like those associated with movement, are activated only when necessary. As a result, this indicates that CPGs could be pacemaker-driven or network-driven [22]. Numerous CPGs have been detected in the spinal cord [23]: the neural circuits responsible for respiration are situated in the brainstem, while those regulating locomotion are in the spinal cord [24]. CPG networks are intrinsically versatile, since they may generate many variations of the same behavior and can be coordinated in diverse ways to elicit distinct patterns [22]. The concept of CPGs was first introduced in the early 20th century by the Nobel laureates Charles Sherrington and Thomas Graham Brown as a result of their pioneering research on locomotion. Subsequent animal studies in lobster [25] and investigations in other vertebrates in lamprey and xenopus tadpole [21,26] have significantly expanded our understanding of these neural circuits and their regulatory mechanisms across various physiological systems, including respiration [27]. Focusing on the regulation of respiratory mechanisms, research has demonstrated that the R-CPG comprises intricate neuronal networks and pathways that are responsible for orchestrating the rhythmic patterns of breathing [26,27]. Any disruption or injury to these pathways can severely impair normal respiratory function, highlighting the critical role of the R-CPG in maintaining effective breathing [28].

### 2.2. Neuroanatomical Structure and Function of R-CPG

**Figure 1:**
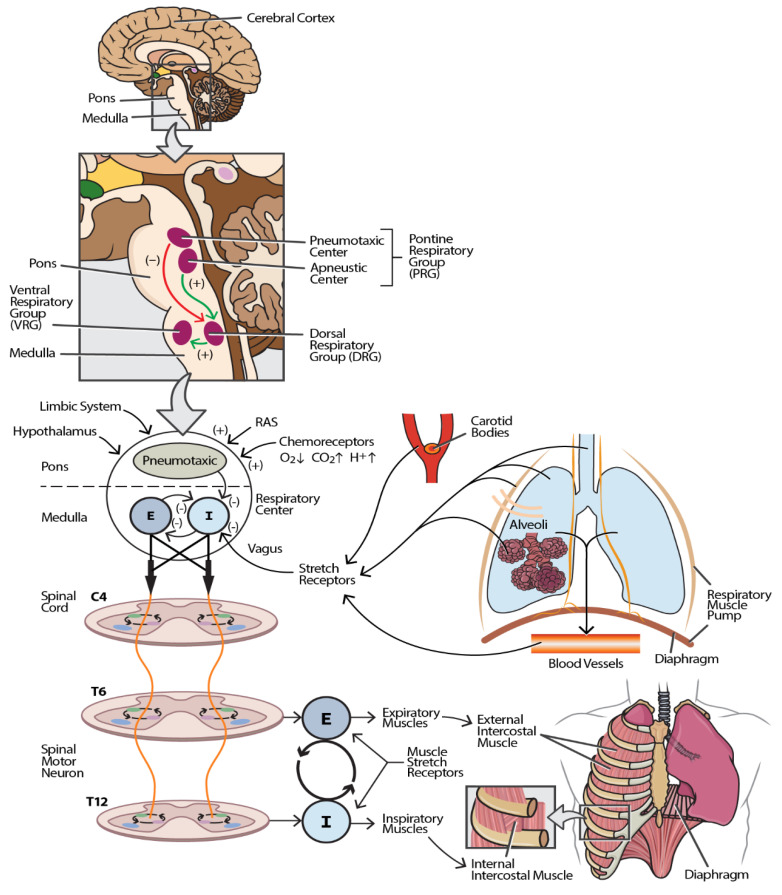
Neuroanatomical and physiological framework of the respiratory central pattern generator (R-CPG). This schematic illustrates the major components and pathways constituting the R-CPG. The inset box enumerates the anatomical and functional elements of the R-CPG, while the main figure depicts their spatial relationships and signaling pathways. The afferent input to the respiratory center (located in the pons and medulla) originates from multiple sources, including respiratory and limb muscles, joint proprioceptors, peripheral receptors in blood vessels, pulmonary stretch receptors (in the lung parenchyma and alveoli), chemoreceptors in the carotid bodies and aortic arch (via the vagus nerve), and central inputs from the reticular activating system (RAS), limbic system, and hypothalamus via spinal and cortical projections. These inputs modulate respiratory neuronal activity by exerting excitatory or inhibitory influences. The efferent output from the respiratory center is conveyed through corticospinal and bulbospinal tracts to spinal motor neurons, which innervate the muscles of respiration. Inspiratory and expiratory motor commands are transmitted to the corresponding muscle groups, orchestrating the rhythmic pattern of breathing. Excitatory pathways are indicated by green arrows (+), while inhibitory pathways are shown as red arrows (−). All the components are functionally interconnected, and disruption at any level may result in respiratory dysfunction. Abbreviations: I—inspiratory impulse; E—expiratory impulse.

Figure 1 shows that the R-CPG comprises several neuroanatomical structures that coordinate voluntary and involuntary control of breathing. The cerebral cortex, located in the cerebral hemispheres, regulates voluntary respiratory control, enabling the conscious modulation of breathing patterns [27]. The hypothalamus, situated in the forebrain, integrates the sensory input related to pain and emotional stimuli, influencing the respiratory activity accordingly [29]. The primary respiratory centers are in the brainstem, specifically within the pons and medulla. These centers include the dorsal respiratory group (DRG), which is responsible for inspiratory control, and the ventral respiratory group (VRG), which facilitates forced expiration. Additionally, the pneumotaxic center modulates the rate and pattern of breathing [30]. The brainstem respiratory centers serve as critical relay sites for all the R-CPG components, facilitating neural integration and synaptic transmission [31]. Central chemoreceptors, located on the ventral surface of the medulla near the ventral parafacial nucleus, are sensitive to changes in carbon dioxide and hydrogen ion concentrations, playing a vital role in maintaining homeostatic respiratory responses [32]. Peripheral chemoreceptors, found in the aortic and carotid bodies, respond primarily to fluctuations in oxygen levels and, to a lesser extent, carbon dioxide and hydrogen ion concentrations [33]. Sensory receptors within the thoracic musculature, including slowly adapting stretch receptors and muscle spindles located in the chest wall, respiratory muscles, airways, and bronchioles, provide critical feedback on lung stretch and volume. These mechanoreceptors transmit information via vagal afferent fibers, contributing to the structural and functional framework of the R-CPG [34].

Damage to any component of the respiratory neural circuitry or its associated pathways can result in significant respiratory control dysfunction. SCI affecting levels from the upper cervical (C2–C3) to the lumbar plexus can cause respiratory impairments of varying severity [10]. Specifically, injuries at the upper cervical level disrupt the bulbospinal tract, which conveys signals from the cerebral cortex to the cervical spinal cord neurons. This disruption interrupts the connection between the descending pathways and lower motor neurons that innervate the diaphragm through efferent nerve fibers [35]. Injuries to the thoracic and lumbar spinal segments compromise the innervation of the thoraco-abdominal muscles, severely affecting respiratory muscle function and breathing mechanics [36]. Furthermore, damage to the respiratory motor cortex and brainstem respiratory centers, and abnormalities in oxygen, carbon dioxide, and hydrogen ion levels—along with dysfunction in pulmonary stretch and irritant receptors—can lead to respiratory impairments and compromised ventilatory control [27].

A comprehensive understanding of the neuroanatomical and physiological framework of the R-CPG is essential for elucidating respiratory pathologies that arise from damage to its components or disruptions in their neural pathways. The intricate neural circuitry of the R-CPG meticulously regulates ventilation, maintaining precise control over respiratory functions. While various CPGs are pre-formed, they exhibit adaptability; some reach the full functional capacity at birth, while others mature through acquired sensory and motor inputs [26]. Understanding the R-CPG and its inherent adaptability offers promising avenues for developing novel interventional strategies aimed at inducing neuroplastic changes by targeting its diverse components. It is hypothesized that a combined approach, involving the activation of multiple elements within the R-CPG, could result in more pronounced functional improvements. The rhythmic nature of the respiratory system further supports the potential for studies employing periodic or intermittent stimulatory inputs to enhance sustained neural plasticity, thereby reinforcing each respiratory cycle. This concept aligns with findings from locomotor studies, where the simultaneous stimulation of multiple targets within CPGs has been shown to augment neuroplasticity and improve functional outcomes [20].

### 2.3. Respiratory Neuron and Spinal Locomotor Generators

Breathing continuously adapts to environmental, metabolic, and behavioral changes through responses to various sensory inputs, including afferent feedback from muscles [37]. Multiple animal studies in rabbits and cats have demonstrated that afferent sensory information from rhythmically moving limbs plays an integral role in modulating breathing patterns [38]. Shevtsova et al. [39] highlighted the modulation of respiration by the peripheral musculoskeletal system, emphasizing the overlapping interactions between locomotor and respiratory neuronal circuits and the influence of lower limb muscle afferents on cardiorespiratory activation during exercise. Numerous experimental investigations in rats have shown that locomotion is functionally connected to respiration through the linkage of CPGs for stepping and breathing [40]. This connection is often attributed to the rhythmic activation of sensory inputs from the limbs during muscular contraction, which modulates respiratory CPG activity [41].

While the effects of peripheral afferents on locomotor–respiratory coupling are well-documented, central mechanisms also play a significant role in the interaction between locomotion and the respiratory system. Early studies by Krogh and Lindhard [42] highlighted the involvement of the cerebral cortex and subcortical structures in activating locomotor–respiratory circuits. Subsequent research identified the mesencephalic locomotor region as a critical area influencing both locomotion and respiration [43]. Additionally, Viala and colleagues [44,45] demonstrated direct interactions between spinal locomotor networks and brainstem respiratory CPGs, indicating a functional connection between spinal locomotor and respiratory neuronal circuits. Le Gal et al. [46] further explored the activation of muscle afferents in experimental settings in rats, examining inputs from limb muscles to the respiratory system through methods such as nerve, muscle, or dorsal root electrical stimulation, as well as active or passive limb movement or stretch [46]. Two significant impacts on respiration have been observed during the experimental stimulation of limb muscle afferents: entrainment of breathing and hyperpnea. The entrainment of breathing involves the respiratory rhythm resetting to follow the frequency of stimuli supplied to muscle afferents [40], while hyperpnea is characterized by an increased breathing frequency and tidal volume [37]. Entrainment is closely associated with locomotor–respiratory coupling, whereas hyperpnea is more related to general physical activity. These respiratory responses are thought to be mediated by both supraspinal and spinal mechanisms [46].

### 2.4. Respiratory Neuroplasticity After SCI

Randleman and colleagues defined neuroplasticity as the long-term structural and/or functional changes within neural networks or the behaviors they support. These changes often occur in response to various stimuli, such as traumatic injury or degenerative disease [2,47]. Enhanced activity within these newly formed neural networks can further promote augmented plasticity, a phenomenon observed in individuals with SCI [48,49]. Neuroplasticity has been extensively documented in both experimental and clinical studies [50]. Most of these studies demonstrate the nervous system’s capacity to adapt and reorganize following the disruption of neural pathways, as observed in SCI [49]. Fawcett et al. [51] noted that spontaneous recovery occurs to some extent in nearly all individuals with SCI. This suggests that while the regeneration of damaged axons may be limited, plastic alterations in intact ascending and descending pathways are possible [52,53]. The targeted conditioning of disrupted neuronal circuits to enhance plasticity can lead to significant functional improvements, offering promising avenues for therapeutic intervention in respiratory rehabilitation following SCI [49].

## 3. Respiratory Neurorehabilitation After SCI

### 3.1. Respiratory Rehabilitation Approaches

Regular exercise has been shown to promote neuronal survival and differentiation in the cortex by increasing blood flow, inducing angiogenesis, and elevating neurotrophic factors [54]. For instance, physical activity enhances the release of brain-derived neurotrophic factor (BDNF), which supports neuronal health and synaptic plasticity [55]. However, limited studies have focused on the effect of exercise alone on axon regeneration. Early investigations suggested that exercise has only a modest impact on axon regeneration across the lesion site and may not be sufficient to induce robust recovery [56,57,58]. Moreover, for any therapy to be translatable to clinical practice, it must include a rehabilitation component, as this forms a part of the standard of care [59]. Evidence-based practices now widely agree that successful SCI management should incorporate activity-based therapy (ABT) to promote functional recovery and effectively enhance guided neuroplasticity [49]. ABT focuses on activating the neuromuscular system below the level of injury, aiming to retrain the nervous system to recover specific motor functions [60].

While rehabilitative interventions have shown promising results in neuromodulation for improving function in targeted areas, pulmonary rehabilitative approaches are increasingly aligning with these strategies [61]. Pulmonary rehabilitation programs have demonstrated efficacy in enhancing exercise capacity and health-related quality of life in patients with chronic respiratory conditions, underscoring the adaptability of the R-CPG to therapeutic interventions [62]. Additionally, studies indicate that pulmonary rehabilitation can induce neuroplastic changes, contributing to improved respiratory outcomes [63].

Respiratory rehabilitation often incorporates respiratory muscle training (RMT), either as a standalone intervention or in combination with pharmacological and/or surgical approaches, such as scES [64]. RMT aims to strengthen and improve the endurance of respiratory muscles, thereby enhancing pulmonary function and reducing respiratory complications in individuals with SCI [65]. When combined with other therapeutic modalities like scES, RMT may further optimize respiratory outcomes by facilitating neuroplastic changes and enhancing the motor control of breathing [66]. Despite its effectiveness, patients frequently report that RMT can be time-consuming, monotonous, and lacks immediate perceived benefits. Furthermore, respiratory performance tends to decline once the training is discontinued [67]. Similarly, pharmacological treatments often result in side effects such as fatigue, which can impair alertness, concentration, and memory, ultimately affecting rehabilitation success rates [15]. In contrast, scES is an emerging technique that has been reported as safe, effective, and FDA-approved, offering a promising alternative for respiratory neurorehabilitation compared with traditional management approaches. However, further research, particularly in larger animal-like swine and human models, is needed to confirm its viability for addressing respiratory deficits following SCI [14]. Unlike traditional pharmacological and physical interventions that typically target a single autonomic function, scES has the potential to address multiple facets of the complex, multimodal dysfunctions associated with SCI [68].

### 3.2. Respiratory Muscle Training (RMT)

RMT is a rehabilitative intervention designed to promote respiratory performance associated with neuroplastic changes [69]. It employs techniques such as impedance load and threshold pressure load to enhance respiratory muscle strength and endurance, targeting inspiratory, expiratory, or both muscle groups [70,71]. Studies conducted in healthy individuals and athletes have demonstrated that RMT improves cardiopulmonary function and optimizes motor performance [72]. Moreover, several studies have suggested that RMT can enhance pulmonary function in individuals with SCI [73,74]. In a systematic review and meta-analysis, Zhang et al. [75] analyzed pulmonary function test results before and after RMT interventions, demonstrating significant improvements. Beyond pulmonary function, RMT has also been associated with positive effects on exercise performance and muscle strength. For instance, peak oxygen consumption during an incremental maximal arm-cranking exercise test improved significantly by 11% following RMT [73]. Additionally, Mueller et al. [76] reported a 55% increase in pectoral muscle strength after RMT, whereas the control group exhibited no change. Furthermore, Houtte et al. [77] noted that patients experienced easier breathing during training, which contributed to an improved quality of life.

### 3.3. Mechanism of Exercise-Induced Neuroplasticity in Motor Recovery

Neuroplasticity after SCI is well supported by evidence from skeletal muscle rehabilitation through locomotion and targeted exercise regimens. Using this as proof of principle, neuroplasticity can also be examined in the context of exercise and respiratory function. Substantial evidence indicates that exercise facilitates motor recovery after SCI through multiple mechanisms. These include: (a) modification of the injury environment, where exercise can alter local conditions at the injury site by reducing inhibitory factors and promoting conditions that are conducive to healing [78]; (b) promotion of axonal sprouting, where physical activity encourages the growth of new axonal branches from both local spinal networks and intact descending pathways, aiding in the re-establishment of neural circuits [79]; and (c) synaptic and ionic plasticity, where exercise induces changes at the synaptic level, enhancing neuronal communication and modifying ionic balances that affect neuronal excitability [80]. These adaptations collectively contribute to improved motor function, underscoring the therapeutic potential of task-specific rehabilitation in functional recovery after chronic SCI [81,82].

Anatomical neuroplasticity in response to exercise after SCI includes axonal growth, regeneration through the lesion core, the sprouting of spared neural tissue, and the formation of novel relays. Additionally, exercise promotes synaptogenesis and strengthens existing neural pathways, leading to spinal cord connectivity remodeling [81]. Forms of exercise, such as treadmill training or wheel running, have been shown to enhance injury-induced axonal sprouting [40,83]. Although no single mechanism has been attributed to these changes, supraspinal pathways have been identified as key contributors. Serotonin is frequently studied due to its significant role in supraspinal modulation. Beyond exercise-induced changes in the expression of spinal 5-HT receptors, plasticity within supraspinal serotonergic projections spared after incomplete SCI has also been observed. Serotonergic projections from the raphe nuclei regulate motoneuron activity in the ventral horn of the spinal cord. Movement, such as stepping, has been shown to improve through increased serotonergic activity with exogenous serotonergic agonists, as demonstrated in animal models [84,85,86]. Additionally, the presence of 5-HT fibers and terminals in the spinal cord strongly correlates with the recovery of locomotor function following injury [87,88].

Exercise has been shown to enhance 5-HT fiber outgrowth, increase the number of 5-HT fibers, and promote the formation of presynaptic terminals around motoneurons in the ventral horn caudal to an incomplete SCI. These adaptations are linked to functional recovery, including improved locomotion and reaching/grasping capabilities [89,90,91]. Exercise-induced elevations in BDNF are likely involved, as BDNF has been associated with structural changes in the lumbar spinal cord following SCI, including the enhanced sprouting of serotonergic axons [90]. Long descending supraspinal pathways are particularly vulnerable to SCI, necessitating significant supraspinal rewiring at a distance from the injury, which is now recognized as a critical component of recovery [92,93]. Following partial SCI, various detour pathways have been identified that transmit supraspinal locomotor commands across the lesion site. Exercise has been shown to contribute significantly to the corticospinal and propriospinal pathway [94] and the corticospinal and reticulospinal pathway [95], demonstrating its role in promoting neural plasticity and functional recovery.

The crossed-phrenic phenomenon [94] serves as a historical example of respiratory flexibility in SCI. Although an injury at this level initially paralyzes the ipsilateral hemidiaphragm, Porter et al. [96] demonstrated that the transection of the contralateral phrenic nerve, which paralyzes both hemidiaphragms, subsequently activated bulbospinal axons that crossed the spinal midline (decussated) below the C2 level to innervate the phrenic motor pool. This was further substantiated by a cross-correlational analysis of phrenic nerve recordings, which confirmed that post-injury function was mediated via bulbospinal pathways [97]. These recordings also suggested a gradual recruitment of spinal interneurons into the damaged phrenic network, potentially contributing to functional plasticity.

Additional evidence of supraspinal plasticity has been demonstrated through sprouting monosynaptic respiratory bulbospinal connections [98,99] and increased serotonergic activity in respiratory centers [100,101]. While these anatomical studies have largely focused on spinal cord neural pathways, respiratory flexibility is a phenomenon observed throughout the neural axis. Respiratory neuroplasticity extends across the CNS, encompassing the brain, brainstem, spinal cord, peripheral nervous system, spinal afferents [102,103], and respiratory musculature [54,104,105]. Identifying and enhancing this anatomical plasticity is crucial for improving respiratory recovery following SCI.

There is also strong evidence of spontaneous respiratory plasticity in the spinal cord, characterized by the formation of novel polysynaptic connections with phrenic motoneurons via cervical spinal interneurons [97,106]. Studies have shown that BDNF upregulation occurs within the phrenic motor neuron pool, playing a key role in enhancing morphological plasticity within the respiratory circuit [107,108]. The activation of normally dormant contralateral respiratory bulbospinal circuits that cross the spinal midline below the lesion is a hallmark of plasticity, with functional restoration occurring in response to respiratory stressors, such as asphyxia, hypoxia, hypercapnia, or contralateral phrenicotomy [94]. In contrast, neural compensation is characterized by altered (e.g., increased) activity in non-injured respiratory circuits and muscles, enabling adaptive function to compensate for post-injury impairments [109].

## 4. Neuromodulation for Respiratory Recovery After SCI

### 4.1. Spinal Cord Epidural Stimulation (scES)

scES was initially employed in the treatment of neuropathic pain in individuals with SCI [110]. More recently, its applications have expanded to include the modulation of motor, sensory, and autonomic functions [111,112]. Although research on the effects of scES on the respiratory system is still in its early stages, the preliminary findings indicate its potential to improve respiratory function [8]. scES targeting the lumbosacral spinal cord has been clinically examined for restoring motor function following SCI, utilizing commercially available devices such as a surgically implantable 16-contact epidural electrode array (5-6-5 Specify, Medtronic, Minneapolis, MN, USA) paired with a rechargeable neurostimulator (RestoreAdvanced, Medtronic, Minneapolis, MN, USA) [112]. These epidural electrodes are quick to implant and have a strong safety profile [82].

The growing interest in scES has led to numerous studies exploring its applications beyond pain relief and spasticity control, focusing on activating spinal circuitry to restore voluntary and involuntary movement and improve posture control after SCI [113]. The technological framework and clinical experience with scES suggest significant potential in enhancing respiratory function, as well as other autonomic systems, including bowel, bladder, sexual, and cardiovascular functions [114]. Over the past decades, scES paradigms have demonstrated promise in evoking respiratory function by activating inspiratory intercostal muscles, with or without synergistic diaphragm contraction [66]. A fully implanted neuroprosthesis would ideally offer individuals with SCI improvements in comfort, aesthetics, and quality of life [115]. Primarily used in pain and spasticity management, fully implanted scES has been a recognized, practical, and safe neuromodulation technique for nearly 50 years [116]. Consequently, epidural neural interface technology is now relatively advanced and has undergone extensive clinical evaluation, with various FDA-approved and commercially available devices [117].

Studies have shown that scES of the lumbosacral spinal cord, along with the recruitment of upper-limb motor neurons, enables participants to make small voluntary leg or arm movements and voluntary trunk muscle contractions during stimulation [118,119]. Notably, muscle activation has been observed prior to any formal training in these individuals. When stimulation was combined with daily standing, stepping, and voluntary exercises, participants were able to generate graded force and maintain contractions on verbal command during full leg flexion exercises [66]. Given the complexity of upper limb movements and the loss of motor neurons following cervical SCI, restoring upper limb function with scES is more challenging compared with lower limb function [120]. However, a case study involving two individuals with AIS B cervical SCI demonstrated improvements in grip strength and motor scores after just one week of scES [121], highlighting its potential in upper limb motor recovery.

Advancements in epidural electrode technology, featuring multiple independent contacts across several spinal segments, allow for the delivery of intricate stimulation patterns targeting diverse motor circuits, thereby enhancing the recovery potential [122]. scES has also been shown to improve trunk stability, with the immediate restoration of trunk control observed in seated positions, even before the initiation of therapeutic training [123]. An enhanced seated reaching performance was noted in two individuals with chronic AIS A SCI during stimulation [124]. Herrity et al. [125] provided proof-of-principle evidence that scES targeting parasympathetic outflow to the bladder could significantly improve the voiding efficiency, potentially through the modulation of the detrusor contraction force and external urethral sphincter relaxation.

Harkema et al. [112] reported the first-in-human application of scES in a person with motor-complete SCI. Among four individuals (AIS B) who received ongoing activity-based interventions over a 44-month period without continuous stimulation, progressive functional improvements were observed, suggesting sustained neural adaptations resulting from long-term activity-based training combined with periodic scES. An independent replication study demonstrated the improved volitional control of motor functions with scES in a participant with AIS A SCI, further supporting its potential to enhance functional outcomes. This participant also experienced notable improvements in bladder, bowel, sexual function, and temperature regulation, including voluntary bladder voiding with minimal residual volume and enhanced sexual performance [82]. Harkema et al. [82] detailed the effects of scES on voluntary movement, standing, and assisted stepping after motor-complete paraplegia. They demonstrated that scES enabled the human lumbosacral spinal circuitry to support full weight-bearing standing when the stimulation parameters were optimized for standing and coupled with bilateral proprioceptive input. When stimulation was adjusted for stepping, locomotor-like patterns emerged, suggesting improved spatiotemporal neuromodulation mimicking natural motoneuron activation during locomotion.

Further research by this group revealed that neuronal networks in the lumbosacral segments could be reactivated into functional states, allowing these circuits to detect sensory input and contribute to neural control, suggesting that the task-specific stimulation training may reactivate dormant brain networks or promote neuroplasticity and offering a viable strategy for functional rehabilitation after severe paralysis [126]. Rejc et al. [127] demonstrated in a case series of four individuals with motor-complete SCI that stand training combined with lumbosacral scES significantly improved motor function, enabling full body weight standing with minimal self-assistance for balance and knee extension. Two participants with AIS A SCI were able to stand independently without external support for hip extension. It is believed that a combinational approach involving scES and activity-based training may yield greater benefits than either intervention alone [68]. This combination has been shown to improve the resting metabolic rate, peak oxygen consumption, and thermoregulatory function [68]. Additionally, the integration of locomotor training with scES has been associated with enhanced oxygen consumption, cardiac output, and lung ventilation. It was assumed that combined rehabilitation strategies may affect supraspinal inputs and sensory feedback for both voluntary sensorimotor and autonomic control [68,128], which all can affect the respiratory function.

### 4.2. Mechanism of scES in Inducing Respiratory Neuroplastic Changes

Lower thoracic scES experiments have typically resulted in local motor root recruitment, eliciting segmental short-latency combined action potentials (CAPs) from the surrounding nerve roots [1,129]. These alterations are likely due to direct depolarization and immediate electrical field effects, as sufficiently high current amplitudes may recruit nerve roots across multiple spinal levels in either direction through rapid current propagation (e.g., across two or three spinal levels, depending on the stimulus intensity) [130]. Notably, the stimulus response curve initially demonstrated a dramatic rise in evoked airway pressure (Paw) from 0 to 15 mA before plateauing at higher amplitudes [131]. scES is well-known for its ability to modulate intrinsic spinal pathways and interneuronal connections, generating both promotive and inhibitory effects on the motor output. When sufficiently activated, these circuits can mediate extensive and remote network effects, including the modulation of functionally related regions of the nervous system distant from the stimulus source [82].

In animal studies in dogs, stimulation at the T9/10 spinal level evoked local-segmental short-latency CAPs at the T9-10 motor roots and long-latency (>3.6 ms) CAPs at the T11-L2 motor roots, suggesting the indirect recruitment of remote caudal motor roots [66]. The recruitment of caudal motor roots significantly contributed to the stimulation-elicited expiratory performance. Sectioning the T8–T10 motor roots markedly affected the elicited expiratory pressures, whereas eliminating distant long-latency CAPs by either T11-L2 root sectioning or dorsal column sectioning led to the most significant reduction in stimulus-elicited Paw, with reductions of up to 60–80% from baseline [132]. Further sectioning of the lateral and ventral funiculi resulted in additional decreases of 16% and 12%, respectively, suggesting that these fiber tracts (including spinocerebellar, corticospinal, rubrospinal, and/or propriospinal pathways) are involved in mediating distant motor network effects [13]. These findings align with existing research supporting the use of scES to enhance breathing and limb motor performance. The modulation of afferent dorsal root fibers via stimulation may contribute to these functional network effects, possibly through mono- or polysynaptic integration at the dorsal column level and/or complex interneuronal networks spanning multiple spinal segments. This provides evidence that low-frequency stimulation (i.e., 10–100 Hz) selectively activates large, myelinated afferents in the spinal roots, particularly proprioceptive primary afferents [133,134].

Experimental models in rats indicate that the sensory activation of other spinal neurons, with inputs near the threshold to stimulate afferent fibers, is unlikely [134,135]. Thus, a sub-threshold level activation approach is often employed. There is substantial evidence that stimulating proprioceptive sensory fibers can lead to both the immediate and long-term modulation of spinal motor reflexes [136,137]. Because inadequate modulation of reflex excitability impairs optimal functional activity, improved modulation is associated with enhanced function. This principle underlies a key mechanism of rehabilitation following injury, where strengthening connectivity through synaptic augmentation is believed to facilitate recovery [138].

Ongoing research is investigating the potential to customize stimulation patterns to activate specific spinal circuits at precise timings, based on the demands of a given motor task [81]. Advances in multi-electrode stimulation technology and the ability to generate diverse stimulus patterns provide a promising opportunity for individuals with SCI to tailor stimulation protocols to their unique rehabilitation needs. Additional evidence, primarily from diffusion tensor imaging studies [56], suggests that the frequent activation of specific neural networks enhances brain connectivity. While the exact mechanisms remain unclear, research indicates that neuronal activity influences myelination patterns in the central nervous system (CNS), as observed in corpus callosal pathways following the acquisition of complex motor skills [57]. After the formation of new neural connections and the reestablishment of excitability in pathways damaged by SCI, sustained activity within these circuits reinforces synaptic strength and connectivity [80]. This can be achieved through carefully designed training regimens, including structured scES protocols, rehabilitative training, or a combination of both. These approaches are proposed to facilitate further adaptive neuroplastic changes, reinforcing motor function and respiratory control [82].

## 5. Other Methods Under Consideration for Improving Respiratory Function/Circuitry

### 5.1. Spinal Cord Transcutaneous Stimulation (scTS)

scTS is a non-invasive neuromodulation approach that specifically targets the spinal cord [139,140]. It has demonstrated potential in facilitating the recovery of functional and motor strength in the upper [141,142] and lower limbs [143,144], as well as improving trunk stability [145,146,147] in individuals with SCI. Studies indicate that scTS promotes sensorimotor recovery [141,142,143], with outcomes further enhanced when combined with physical training [18,145,148]. Although some overlap exists between the mechanisms of action of scTS and physical training, their distinct and potentially synergistic processes may lead to a more effective reorganization of neuronal circuits [18]. The precise underlying mechanism remains unclear, necessitating further research to validate these findings.

Additionally, scTS has shown a significant impact on cervical SCI, with improvements in breathing and coughing ability [149]. Previous research has demonstrated that robot-assisted locomotor training in chronic SCI led to increased oxygen consumption, minute ventilation, and pulmonary ventilation, contributing to respiratory muscle activation [150]. A recent study revealed that scTS, in conjunction with inspiratory muscle training, enhanced inspiratory and expiratory muscle strength and pulmonary vital capacity, while also improving breathlessness and hypophonia [18]. These findings support the potential of scTS as an alternative treatment to facilitate recovery from respiratory deficits following SCI.

Further, scTS has been documented to stimulate the same neural networks as scES [151,152] and may elicit comparable outcomes [149]. Unlike scES, which requires an invasive neurosurgical procedure with potential complications, scTS provides a non-invasive and safer alternative. In Table 1, a summary of scES and scTS have been presented as clinical evidence of these approaches. Additionally, scTS electrodes can be repositioned along the spinal cord to simultaneously target multiple organ systems [149]. The use of scTS in clinical studies and among individuals with SCI is expanding [151,153,154]. Future research is warranted to establish more conclusive evidence supporting scTS as an effective therapy for functional and respiratory rehabilitation following SCI.

### 5.2. Limb Muscle Stimulation as a Therapy to Treat Respiratory Dysfunction Following SCI

Therapies incorporating limb muscle stimulation have shown promise in addressing respiratory dysfunction following SCI. Shevtsova et al. [39] highlighted the potential of locomotor training-based therapies in treating respiratory issues across various medical conditions. For example, incorporating upper limb exercises into pulmonary rehabilitation in patients with chronic obstructive pulmonary disease has been associated with reduced episodes of dyspnea [62]. Similarly, exercise programs targeting the upper extremities have significantly improved respiratory function in individuals with multiple sclerosis [158]. Strengthening exercises for the upper limbs have also demonstrated benefits in respiration for children with cerebral palsy and patients who have experienced stroke. While these studies primarily focused on enhancing muscular strength and normalizing vertebral alignment, the activation of afferent pathways from working muscles may also play a crucial role in respiratory improvement [159,160].

As anticipated, lower extremity activation can also promote respiratory recovery. Daily electrical stimulation of leg muscles in patients in the intensive care unit has been shown to reduce the duration of assisted ventilation, facilitating earlier weaning compared with non-stimulated groups [161]. Additionally, vibratory stimulation over hand and foot proprioceptors decreased the number of apneic and hypoxic episodes in premature infants (less than 34 weeks’ gestation), suggesting that limb muscle stimulation can enhance breathing [162]. Clinical studies indicate that limb muscle stimulation may be beneficial in respiratory rehabilitation in individuals with SCI. Repetitive activation of the impaired respiratory system through peripheral muscle stimulation could retrain spared neural networks, enhancing their function post SCI. This activation can occur via supraspinal mechanisms (in cases of incomplete SCI), as well as spinal pathways [82]. Several studies have demonstrated the beneficial effects of lower and upper limb afferent activation on respiratory function in patients with SCI. Rhythmic stimulation of lower extremities through assisted treadmill locomotion has been shown to elicit metabolic responses and increase ventilatory parameters in individuals with chronic complete and incomplete cervical SCI [163]. Regular treadmill training with body weight support, combined with functional electrical stimulation (FES), significantly improved respiratory motor function in patients with cervical and thoracic SCI [164].

Moreover, exercises involving the upper extremities have also been shown to enhance ventilatory function in individuals with thoracic SCI [53]. Given the logistical and economic challenges of treadmill training in tetraplegic individuals, FES of the upper extremities presents a more feasible alternative. This non-invasive approach, utilizing cutaneous electrode placement, requires fewer personnel and can be administered in a home environment. For example, arm-cranking exercises assisted by FES have been employed to enhance respiration in individuals with cervical SCI [165]. Furthermore, combinatorial strategies, such as integrating FES with exercises like bicycling, arm-cranking, or arm-cycling, have demonstrated a greater potential for recovery in individuals with SCI by producing more pronounced effects on cardiorespiratory function [166]. While these clinical studies have yielded promising results, further research is necessary to optimize these strategies for improving ventilation in individuals with SCI. Translating these findings to animal models of SCI may provide deeper insights into the mechanisms and efficacy of limb muscle stimulation in respiratory rehabilitation.

### 5.3. Acute Intermittent Hypoxia (AIH)

AIH has been shown to induce spinal respiratory motor plasticity in animal models in rats, suggesting the potential for enhancing breathing capacity in individuals with SCI [167]. Sutor et al. [168] investigated the effects of a single AIH session on respiratory function in individuals with chronic SCI, finding that AIH increased the maximum inspiratory pressure generation but did not significantly affect other breathing functions. This selective improvement in inspiratory pressure may stem from differences in the capacity for AIH-induced plasticity and neuromodulation within inspiratory motor neuron pools, or from the more preserved innervation of inspiratory muscles compared with expiratory muscles [169].

Hypoxia stimulates the pneumotaxic center in the pons, which activates respiratory neurons in the medulla oblongata. These neurons transmit descending neural fibers through spinal motor neurons to the diaphragm and other respiratory muscles [170]. This pathway suggests that AIH could enhance neuroplasticity in inspiratory motor neurons or residual neural circuits, thereby improving the activation of thoracoabdominal muscles and increasing the maximum inspiratory pressures [171]. These findings support AIH as a potential non-invasive intervention for promoting neuroplasticity in inspiratory motor neurons and improving respiratory function in individuals with SCI.

### 5.4. Vocal Respiratory Training (VRT)

VRT, often combined with music therapy, is utilized to strengthen respiratory muscles and improve lung function [67]. Zhang et al. [172] investigated the therapeutic effects of VRT on respiratory dysfunction in individuals three months post cervical SCI, reporting significant improvements in respiratory function. Furthermore, neuroimaging studies revealed the increased diversification of nerve fiber bundles in the medulla’s respiratory center, including enhancements in nerve fiber number, length, thickness, and density [173]. While singing has been used to train respiratory and vocal muscles in individuals with SCI, showing positive outcomes, these findings suggest that music-based VRT may promote neuroplasticity within respiratory pathways, potentially leading to improved respiratory function [174].

### 5.5. Gene Therapy

Recent advances in gene therapy and targeted delivery techniques have significantly improved our understanding of respiratory motor systems. Gene therapy, which involves introducing genetic material into cells, has gained broad interest as a potential intervention for SCI. Enhanced vector design tools, including plasmids, synthetic or viral vectors, and cell-based treatments, have driven progress in this field. Among these, adeno-associated viruses (AAVs) have become one of the most widely used vector systems due to their adaptability and well-characterized targeting of specific tissues [175].

Mantilla et al. [176] demonstrated the efficacy of AAV-mediated gene transfer in respiratory motoneurons, showing that intramuscular injections of AAVs successfully transduced respiratory motoneurons, while intraspinal injections improved ventilatory function in animal models, such as mice and chickens. These findings suggest that AAV-mediated gene therapy can induce neuroplasticity in respiratory pathways, holding promise for therapeutic applications in conditions affecting respiratory function [177].

## 6. Conclusions

Rehabilitative strategies targeting one or multiple components of the R-CPG, tailored to each individual with SCI, have the potential to enhance neuroplasticity, promote the formation of new neuronal connections, and facilitate synaptic modulation. These processes may lead to significant functional improvements and a reduction in respiratory morbidity and mortality. Further research is necessary to develop and evaluate regimens that integrate respiratory motor training with scES or scTS to improve respiratory motor function and strengthen the thoracoabdominal musculature. Such strategies could enhance the cough reflex, improve the clearance of respiratory and oral secretions, and mitigate respiratory infections, ultimately contributing to a better quality of life for individuals with SCI and their families, while reducing the burden on healthcare systems. This review has provided an overview of the current literature on neuromodulation strategies, their mechanisms of action, and evidence of efficacy, emphasizing the potential of combining interventions to leverage R-CPG-driven plasticity and augment respiratory function. In addition, a summary of different therapeutic approaches has been presented to assist clinicians and researchers in understanding its potential in clinical practice (Table 2). However, there remains a critical need for large-scale, randomized clinical trials to determine the long-term efficacy and optimal neurorehabilitation approaches tailored to the specific needs of individuals with SCI.

## Figures and Tables

**Table 1:** Summary of clinical evidence of two neuromodulation approaches for respiratory improvement.

Author	Subjects & LOI	Mechanism	Outcomes	Clinical Limitations
Spinal cord epidural stimulation (scES)				
Kandhari et al. [155]; DiMarco et al. [156]; [157]	3 males-C2–C4; 2 males-C2–C4; 10 males-C4–T1	scES modulates intrinsic spinal pathways and interneuronal connections exerting either facilitative or inhibitory effects on the motor output, possibly via mono- or polysynaptic integration at the level of the dorsal column and/or via complex interneuronal networks.	Showed evoked muscle activation of physiological breathing, increasing the inspiratory–expiratory effort, and airway pressure generation. In addition, scES demonstrated potential in evoking respiratory function by recruiting inspiratory intercostal muscles with improved maximum inspiratory pressure, maximum expiratory pressure, total lung capacity, etc.	Much of the progress in clinical applications has relied on the early discoveries made in animal models lacking human research. While scES is promising, it requires a highly invasive neurosurgical procedure, which is expectedly associated with significant morbidity. There could be the potential for adverse electrical interactions due to the high stimulus intensities required to activate each muscle group.
Spinal cord transcutaneous stimulation (scTS)				
Kumru et al. [18]; Gad et al. [149]; Tharu et al. [154]	10 males/1 female-C4–C7; 1 male-C5; 1 female-C4	Activation of inaccessible neuronal networks of the spinal cord likely including the recruitment of afferent fibers (large–medium) in the posterior root in order to elevate spinal network excitability	Excites neuronal circuits and facilitates functional recovery, including autonomic functions. It showed improved respiratory function with significant improvements in maximum inspiratory pressure, maximum expiratory pressure, and forced vital capacity (FVC), as well as in subjective measures of dyspnea. In addition, the placement of electrodes for scTS could target specific segments of the spinal cord and promote the desired functional outcomes.	It is still necessary to explore and understand the optimal parameters of scTS at different segments of the spinal cord. Further research is needed to validate the results and establish the long-term benefits of scTS. Additionally, the mechanisms underlying the action of scTS are partially overlapping, as it is combined with training that may involve different and perhaps synergistic processes, suggesting more clinical trials are needed to understand the efficient reorganization of neural circuits.

Level of injury: LOI.

**Table 2:** Summary of different therapeutic approaches used for respiratory function recovery in SCI.

Author	Intervention	Outcomes	Clinical Limitations	Advantages
Galer [14]; Rejc et al. [111]; Harkema et al. [112]; Darrow et al. [114]; Sayenko et al. [123]	scES	Reactivating neuronal networks in the lumbosacral segments into functional states showed potential in enhancing respiratory function, as well as other autonomic systems, including bowel, bladder, sexual, and cardiovascular functions.	Invasive method, requires surgery, expensive, risk of infection, technical issues, etc. Majority of studies are in animals, therefore research in larger mammals and humans is required to determine safety and feasibility.	Safe, effective, FDA approved, reported to be effective in SCI, traumatic brain injury, stroke, etc.
Leemhuis et al. [15]; Shah et al. [178]; Yang et al. [179]; Li et al. [180]	Pharmacological intervention	Improving the regeneration microenvironment by reducing glial scars, neuronal death, and the overall neuroinflammation process.	Acting mainly on a single mechanism and one target is not sufficient to treat the disease; side effects such as fatigue, which can impair alertness, concentration, and memory, ultimately affect rehabilitation success rates.	Cost-effective, acting mainly on inflammatory processes and alterations in the vascular system due to the trauma.
Inanici et al. [142]; Rath et al. [147]; Tharu et al. [154]; Kumru et al. [18]	scTS	Demonstrated potential in facilitating functional recovery and motor, sensory and recovery of various autonomic functions, including respiratory function.	Some overlap exists between the mechanisms of action of scTS as it is delivered with physical training, bulky equipment, and not suitable for home use; long-term effects still need to be explored.	Non-invasive, could stimulate the same neural networks as scES and produce comparable outcomes. scTS electrodes can be repositioned along the spinal cord to target multiple organ systems.
DiMarco [66]; Tamplin et al. [67]; Verges et al. [73]; Zhang et al. [75]; Van Houtte et al. [77]	RMT	Facilitating neuroplastic changes and enhancing motor control of breathing.	Time-consuming, monotonous, and lacking immediate perceived benefits; respiratory performance tends to decline once training is discontinued.	Strengthens and improves the endurance of respiratory muscles, thereby enhancing pulmonary function and reducing respiratory complications.
Leemhuis et al. [15]; Nagoshi et al. [181]; Dasari et al. [182]; Zhu et al. [183]	Stem cell therapy	Inducing neuroplasticity in respiratory pathways and improving ventilatory function.	Still being developed, efficacy has been questioned due to the contradictory results reported, concerns about the expense of developing adult stem cells, showed poor results, large clinical trials investigating the therapeutic efficacy of stem cell therapy in humans are lacking.	Evidence of nerve generation but not functional recovery, seems to cause no harm and appears to be safe, showing no adverse reactions or side effects, has gained broad interest.

scES: spinal cord epidural stimulation; scTS: spinal cord transcutaneous stimulation; RMT: respiratory muscle training.

## Data Availability

No new data were created or analyzed in this study. Data sharing is not applicable to this article.
